# Responses of Cyanobacterial Crusts and Microbial Communities to Extreme Environments of the Stratosphere

**DOI:** 10.3390/microorganisms10061252

**Published:** 2022-06-19

**Authors:** Qi Li, Chunxiang Hu, Haijian Yang

**Affiliations:** 1 Key Laboratory of Algal Biology, Institute of Hydrobiology, Chinese Academy of Sciences, Wuhan 430072, China; liqi1689@163.com (Q.L.); cxhu@ihb.ac.cn (C.H.); 2 University of Chinese Academy of Sciences, Beijing 100049, China

**Keywords:** stratosphere, cyanobacterial crusts, photosynthetic activity, enzymes activities, microbial community

## Abstract

How microbial communities respond to extreme conditions in the stratosphere remains unclear. To test this effect, cyanobacterial crusts collected from Tengger Desert were mounted to high balloons and briefly exposed (140 min) to high UV irradiation and low temperature in the stratosphere at an altitude of 32 km. Freezing and thawing treatments were simulated in the laboratory in terms of the temperature fluctuations during flight. Microbial community composition was characterized by sequencing at the level of DNA and RNA. After exposure to the stratosphere, the RNA relative abundances of *Kallotenue* and *Longimicrobium* increased by about 2-fold, while those of several dominant cyanobacteria genera changed slightly. The RNA relative abundances of various taxa declined after freezing, but increased after thawing, whereas cyanobacteria exhibited an opposite change trend. The DNA and RNA relative abundances of Nitrososphaeraceae were increased by 1.4~2.3-fold after exposure to the stratosphere or freezing. Exposure to stratospheric environmental conditions had little impact on the total antioxidant capacity, photosynthetic pigment content, and photosynthetic rate, but significantly increased the content of exopolysaccharides by 16%. The three treatments (stratospheric exposure, freezing, and thawing) increased significantly the activities of N-acetyl-β-D-glucosidase (26~30%) and β-glucosidase (14~126%). Our results indicated cyanobacterial crust communities can tolerate exposure to the stratosphere. In the defense process, extracellular organic carbon degradation and transformation play an important role. This study makes the first attempt to explore the response of microbial communities of cyanobacterial crusts to a Mars-like stratospheric extreme environment, which provides a new perspective for studying the space biology of earth communities.

## 1. Introduction

The stratosphere is a natural site to explore the limits to life survival and the protective mechanisms of cells and model systems. In the stratosphere, multiple extreme conditions exist simultaneously, such as extreme aridity (relative humidity of ~23%), low temperature (down to −70 °C), and high UV radiation (UVC up to 860 J m^−2^) [1,2,3]. These conditions are similar to those on the surface of Mars. In recent years, high-altitude aircraft technological advances allow biological experiments to be carried out in the stratosphere [4,5].

Very few microbes have been collected and cultured from the stratosphere, and most collected microbes were spore-forming microorganisms, such as *Bacillus luciferensis* and *Penicillium* sp. [6,7,8,9,10]. Biological exposure experiments have been performed with bacteria, archaea, and fungi to explore the survival potential of microorganisms in the stratospheric environment. The viability of *Bacillus pumilus* endospores was significantly reduced by two orders of magnitude when exposed to sunlight in the stratosphere at 31 km for 2 h with UV radiation as the primary cause of spore damage [11,12]. The exposure of two halophilic archaea to altitudes of 36 km for 1 h resulted in 50% survival of *Halorubrum lacusprofundi* (a cold-adapted species) but only 5% survival of *Halobacterium* sp. NRC-1 (a radiation-tolerant species) [13], implying that low temperature in the stratosphere could be lethal to archaea. The exposure to stratospheric conditions (25 km for 110 min) resulted in a loss of 90% vs. 99% viability in the extremophilic fungi *Naganishia friedmannii* 16LV2 and *Exophiala* sp. 15LV1, respectively [14]. These results indicate that single populations have poor tolerance to the extreme environmental conditions of the stratosphere, with UV radiation and low temperature as the two most important factors affecting microbial survival rates. However, the mechanism underlying complex microbial communities’ response to stratospheric environmental conditions remains largely unknown. The exploration of the responses of microbial communities to stratospheric conditions would not only broaden the scope of space biology from populations to communities but also expand our understanding of the means and the extent of microbial adaption to extreme environmental conditions.

Desert region environments most closely resemble stratospheric conditions in terms of scarce precipitation and intense UV radiation. In these areas, filamentous cyanobacteria and their extracellular polymeric substances bind and cement soil particles, gradually forming composite layers on the soil surface [15,16]. These biological soil composite layers dominated by cyanobacteria are called cyanobacterial crusts (CCs) and comprise a variety of bacteria, archaea, and eukaryotic communities [17,18,19]. Some taxa, especially filamentous cyanobacteria, are UV resistant [20,21,22]. Biological crusts are regarded as ideal model systems because they are easy to manipulate and are sensitive to external environmental changes [23,24]. Therefore, the exposure of CCs to the stratosphere could not only reveal the responses of microbial communities to the extreme environments but also display the changes in their metabolic activity in these environments.

Previous studies have shown that CCs can be adapted to radiation and low temperature through various strategies such as sophisticated niche and antioxidant systems [25,26]. Therefore, we hypothesized that CCs could be adapted to stratospheric environmental conditions with little damage. To verify this hypothesis, we performed stratospheric exposure and low-temperature incubation experiments in this study. Photosynthetic activity and enzyme activity were measured to identify damage. The changes in microbial community composition were examined via 16S rRNA gene (DNA) and 16S rRNA (RNA) sequencing.

## 2. Materials and Methods

### 2.1. Natural Cyanobacterial Crusts Collection

**Figure 1:**
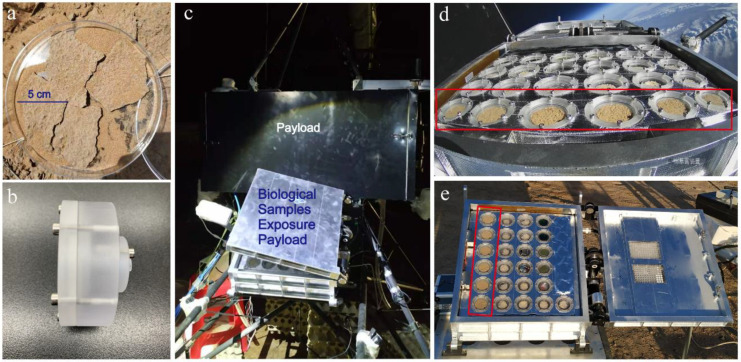
Sample preparation and experimental design. (**a**) Cyanobacterial crusts collected from Shapotou; (**b**) side structure of sample box; (**c**) biological samples exposure payload tilted on the payload; (**d**) cyanobacterial crusts in the stratosphere during light exposure; (**e**) cyanobacterial crusts on the ground. Cyanobacterial crusts are shown in the red wireframe.

Natural cyanobacterial crusts (100% coverage of cyanobacteria, dominated by *Microcoleus*) [27] were collected in August 2019 from the Shapotou Region of Ningxia Hui Autonomous Region, located on the southeastern edge of the Tengger Desert in China (37°32′ N, 105°02′ E; 1339 m elevation). The region has a typical continental monsoon climate with an annual average temperature of 10.0 °C (with temperatures ranging from −25.1 °C to 38.1 °C). The annual average rainfall is approximately 293 mm in this region. To minimize the heterogeneity among samples, the sample collection area (above 2 m × 3 m) was selected to be far away from shrubs and human interference, containing only cyanobacterial crusts. Sterile Petri dishes (15 cm diameter) were put into the soil upside-down, and CCs were peeled off with a sterile spatula (natural thickness is about 2 mm) (Figure 1a). The samples in each sterile Petri dish were divided into four aliquots, corresponding to three treatment groups and a control group as described below. The collected CCs were kept dry at room temperature and immediately transported to the laboratory.

### 2.2. Experiment Design

The crusts were installed into customized sample boxes under sterile conditions. Each sample had a diameter of 3.6 cm and a mass of about 4.2 g. The whole sample box was sealed with a 0.2 μm filter installed at the bottom (Figure 1b and Figure S1). To ensure full light exposure of samples, the fused silica glass (JGS2) was installed on top of the sample box, thus resulting in an uninterrupted UV exposure of 93%. Sample boxes containing crusts were transported to the experimental base in Da Qaidam Town, Qinghai Province (37°74′ N, 95°34′ E; 3182 m elevation).

At the experimental base, the stratospheric exposure (UV + LT) treatment was performed as follows. Eight hours before the zero-pressure high-latitude balloon (volume: 50,000 m^3^) was released, crusts were installed onto the sample plate and put into a refrigerator at 4 °C. Two hours before the balloon departed, the sample plate was installed into a Biological Samples Exposure Payload (BIOSEP, 57 cm × 43 cm × 20 cm, mass 25 kg) with the box cover closed. In the middle of the BIOSEP, two chips were installed to measure light intensity and temperature in real time. The BIOSEP was installed at an angle of 30° from the horizontal plane (Figure 1c). Subsequently, the payload was connected to the balloon; the balloon was filled with helium; the instruments were checked; and the balloon was launched at 5:16 am (Beijing time). The payload reached an altitude of 32 km at 7:15 am, and the BIOSEP was opened to expose CCs to the stratosphere (Figure 1d). After flying horizontally for 140 min, the BIOSEP cover was closed, and the payload was disconnected from the balloon. The payload descended gradually with the recovery parachute and landed at 10:22 am. Recovery crews arrived at the landing site within 5 min. The BIOSEP and sample boxes were not broken. The sample plate was taken out in the shade, placed into a refrigerator at 4 °C, and quickly transported back to the experimental base. The real-time temperature and light intensity of sample boxes were presented in Table S1. The temperature first declined and then increased. From balloon departure time to BIOSEP opening time, the temperature decreased slowly from about 7 °C to −35 °C. From the opening to the closing of the BIOSEP, the temperature slowly increased from about −35 °C to 0 °C.

In order to compare with exposed CCs, we prepared a natural control (CK) at the balloon departure location. For the CK group, CCs were installed with the same model of BIOSEP (Figure 1e), and CK samples were not attached to the balloon, but they were exposed and recovered synchronously with the UV + LT treatment group. The BIOSEP was installed at an angle of 30° from the horizontal plane, without any obstruction in front. Each treatment contained 6 boxes of samples. Three boxes of samples were used for DNA/RNA extraction and physiological and biochemical indicator determination with each box as a parallel. Half sample of each box was used for DNA/RNA extraction, and the remaining half was used for physiological and biochemical indicators determination. The other 3 boxes of samples were used for CO_2_ exchange rate measurement. The samples for DNA/RNA extraction were fully ground with mortars and pestles and passed through 80-mesh sieves, and then they were placed in dry ice. Other samples were placed in a dark icebox (3–6 °C) and quickly transported to the laboratory.

To simulate the effect of a low-temperature environment in the stratosphere on crusts, CCs were subjected to low-temperature freezing (LT-F) and low-temperature thawing (LT-T) treatments in the dark. For the LT-F treatment, CCs were placed into a programmed cryogenic temperature apparatus (Custom BioGenic Systems, Bruce Township, MI, USA) and cooled from 10 °C to −40 °C (at the rate of 0.5 °C min^−1^) within 100 min. For the LT-T treatment, CCs were placed into a glass box connected to a low-temperature thermostat (Shanghai Yiheng Technology Co., Ltd., Shanghai, China) and warmed from −40 °C to 0 °C (at the rate of 0.5 °C min^−1^) within 80 min. There were also 6 boxes of samples for each low-temperature treatment. Three boxes of samples were used for DNA/RNA extraction and physiological and biochemical indicators determination. The other 3 boxes of samples were used for CO_2_ exchange rate measurement.

### 2.3. Physiological and Biochemical Indicators Measurement

To measure the CO_2_ exchange rate, CCs were hydrated with distilled water to saturation and exposed at a light intensity of 40 μmol photos m^−2^ s^−1^ (25 ± 2 °C) with the cycle of hydration and exposure lasting for 6 h [28]. After 6 h of cycle, CCs were respectively exposed to low, medium, and high light intensities (40, 60, and 80 μmol photos m^−2^ s^−1^) for 20 min, and the CO_2_ exchange rate of CCs was measured with a soil carbon release rate device (Yaxinliyi Sci Technol Co. Ltd., Beijing, China). During these measurements, CCs were placed in a transparent closed chamber (volume of 0.164 L). CO_2_ flow rate was automatically detected by the system and converted into μmol CO_2_ m^−2^ s^−1^. The carbon exchange rate under dark and light conditions represented the dark respiration rate (R_dark_) and net photosynthesis rate (Pn) of the CCs, respectively. The gross photosynthesis rate (Pg) was calculated according to R_dark_ and Pn under three light intensities. The chlorophyll fluorescence of CCs was measured with a portable handheld PEA (Hansatech Instruments Ltd., King’s Lynn, UK) after 15 min dark adaptation in order to determine the ratio of variable fluorescence to maximal fluorescence (Fv/Fm). Fv/Fm is an indicator of the maximal photochemical quantum yield, reflecting the maximal light energy conversion efficiency of photosystem II.

To determine physicochemical indicators, CCs were fully ground using mortars and pestles and passed through 80-mesh sieves. The contents of chlorophyll-a (Chl-a) and carotenoids (Car) were measured and calculated according to previously reported methods [29]. The 0.8 g sample was weighed and put into a glass tube. Four milliliters of 100% acetone was added into each tube, and samples were placed in the dark at 4 °C for 24 h. The supernatant was collected by centrifugation, and absorbance values at 663 nm, 490 nm, and 384 nm were measured using a spectrophotometer. The content of bacterial chlorophyll (Bchl) was measured as follows: A 4 mL mixture of acetone and ethanol (at a volume ratio of 7:2) was added into 0.8 g sample and shaken for 2 h at room temperature in the dark. The supernatant was obtained by centrifugation. The absorbance of the supernatant at 771 nm and 747 nm was measured with a spectrophotometer, and the content of Bchl was calculated according to the method reported by Rest and Grigeas [30].

Exopolysaccharides (EPS) were extracted from samples (0.1 g) three times with 0.1 M Na_2_EDTA for 15 min [31]. The volumes of extraction solution were 4 mL, 3 mL, and 3 mL, respectively. The three extracts were pooled, and 1 mL 9% phenol and 5 mL concentrated sulfuric acid were added to 2 mL of the pooled EPS extracts. After 30 min of incubation, the absorbance of the reaction mixture at 485 nm and 750 nm was measured, and the content of EPS was calculated according to the glucose standard curve [32]. The activities of N-acetyl-β-D-glucosidase (S−NAG, 0.1 g), β-glucosidase (S−GC, 0.1 g), ribulose-bisphosphate carboxylase (RuBisCo, 0.1 g), total antioxidant capacity (T−AOC, 0.1 g), and the soil glutathione (GSH, 0.1 g) content were determined using commercially available kits (Suzhou Comin Biotechnology Co., Ltd., Suzhou, China). All the experiments were performed in triplicates for each sample.

### 2.4. Microbial Community Analysis

RNA was extracted in triplicates from ~2.0 g CCs using the RNeasy PowerSoil Total RNA Kit (Qiagen, Hilden, Germany). After RNA extraction, DNA was eluted from the spin filter using the RNeasy Powersoil DNA Elution Kit (Qiagen). Then residual DNA was removed from RNA using the DNase Max Kit (Qiagen). The obtained DNA and RNA were detected by agarose gel electrophoresis and NanoDrop 8000 (Thermo, Waltham, MA, USA). The qualified DNA and RNA were subjected to amplification sequencing by Majorbio Bio-pharm Technology Co., Ltd., (Shanghai, China) on an Illumina MiSeq PE300 platform (Illumina, San Diego, CA, USA). RNA was reverse-transcribed to cDNA using a HiScript 1st Strand cDNA Synthesis Kit (Vazyme Biotech Co., Ltd., Nanjing, China). The V4 region of the bacterial 16S rDNA gene was amplified with primers 338F (5′-ACTCCTACGGGAGGCAGCAG-3′) and 806R (5′-GGACTACHVGGGTWTCTAAT-3′) [33]. The unique barcodes combined with primers were used to distinguish samples. PCR was performed in a 20 μL reaction system containing 0.8 μL of each primer (5 μmol L^−1^), 4 μL of 5 × FastPfu buffer, 2 μL of dNTPs (2.5 mmol L^−1^), 0.4 μL of TransStart FastPfu DNA polymerase (MBI Fermentas, Waltham, MA, USA), and 10 ng of template DNA/cDNA. The PCR procedures were as follows: pre-denaturation at 95 °C for 3 min, followed by 30 cycles of denaturation at 95 °C for 30 s, annealing at 58 °C for 30 s, and extension at 72 °C for 45 s, ending with a final extension at 72 °C for 10 min. All the PCRs were conducted in triplicate. The same conditions and procedures were adopted for archaea PCR amplification using the primers Arch349F (5′-GYGCASCAGKCGMGAAW-3′) and Arch806R (5′-GGACTACVSGGGTATCTAAT-3′) [34]. PCR products from triplicates were pooled and verified by 2% agarose gel electrophoresis. The pooled products were purified using AxyPrep DNA Gel Extraction Kit (Axygen, Hangzhou, China) and quantified with a Quantus™ Fluorometer (Promega, Madison, WI, USA). The purified PCR products from each sample were pooled equimolarly for sequencing.

The sequences were analyzed using the free online Majobri I-Sanger Cloud Platform. All the raw reads were aligned to samples according to different barcodes. Both forward and reverse primers were trimmed. Unqualified sequences were filtered using fastp version 0.20.0 software [35] with a quality score > 20 as the threshold. Paired-end reads with sufficient length (>50 bp) were connected into full-length sequences with at least 10 bp overlap using the FLASH tool [36]. UPARSE was used to remove chimeric sequences and classify sequences into operational taxonomic units (OTUs) at a similarity of 97% [37]. All singleton OTUs were removed. The representative sequences of bacteria were annotated taxonomically against the non-redundant nucleotide database with a confidence cutoff of 0.7. Finally, subsampling was performed to normalize the dataset of the sample based on the lowest number of sequences.

### 2.5. Statistical Analysis

The alpha diversity indices (including Shannon and Chao1) were calculated at the OTU level using the Vegan package in R v4.0.1 (https://cran.r-project.org/src/contrib/Archive/vegan/, accessed on 7 August 2021). The differences in photosynthetic activities, pigment contents, EPS contents, enzyme activities, and alpha diversity indices were analyzed by one-way analysis of variance (ANOVA) based on mean values of three replicates, using SPSS 25.0 software (SPSS Inc., Chicago, IL, USA). Post hoc multiple comparisons were performed using Tukey HSD. An archaeal neighbor-joining phylogenetic tree was constructed using MEGA 7.0 software after computing the evolutionary distances via the maximum composite likelihood method. The confidence limits for the tree topology were estimated using bootstrap analysis with 1000 replications.

## 3. Results

### 3.1. Physiological and Biochemical Changes of Cyanobacterial Crusts

**Figure 2:**
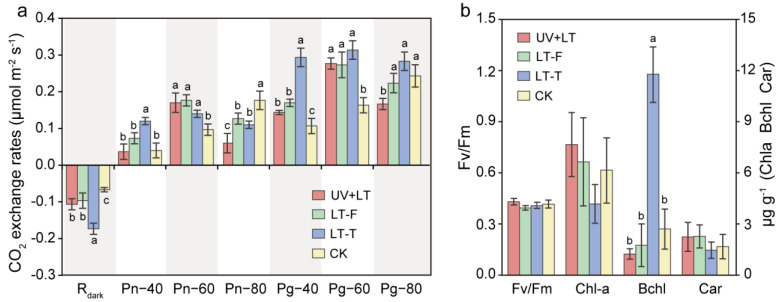
Photosynthetic activity of cyanobacterial crusts. (**a**) The CO_2_ exchange rate of cyanobacterial crusts under different light conditions. R_dark_, the dark respiration rate; Pn−40, Pn−60, and Pn−80 represent net photosynthesis rate under the light intensity of 40 μE, 60 μE, and 80 μE, respectively; Pg−40, Pg−60, and Pg−80 represent gross photosynthesis rate under the light intensity of 40 μE, 60 μE, and 80 μE, respectively. (**b**) Fv/Fm values and content of chlorophyll-a (Chla), bacterial chlorophyll (Bchl), and carotenoids (Car). UV + LT: stratospheric exposure; LT-F: low-temperature freezing; LT-T: low-temperature thawing; CK: natural control. Error bars show mean ± SD and different lowercase letters indicate significant difference (Tukey HSD; *p* < 0.05) between treatments and control (*n* = 3).

R_dark_ was significantly increased under three treatments (F = 26.04, *p* < 0.001), while Pn and Pg were initially increased and then decreased with increasing light intensity (Pn: F = 12.59–26.04, *p* < 0.005; Pg: F = 9.33–65.519, *p* < 0.006) (Figure 2a). The average contents of Chl-a in UV + LT, LT-F, LT-T, and CK groups were 7.66, 6.64, 4.17, and 6.16 μg/g, but there was no significant difference. The Bchl content significantly increased in the LT-T group, but no significant changes were observed in UV + LT and LT-F groups (F = 53.66, *p* < 0.001). None of the treatments revealed a significant change in the content of the anti-stress pigment carotenoids. Fv/Fm value showed no significant difference under the three treatments (Figure 2b). These results implied that the short-term exposure to stratospheric environmental conditions had no substantial impact on the photosynthetic activity of cyanobacterial communities in CCs.

**Figure 3:**
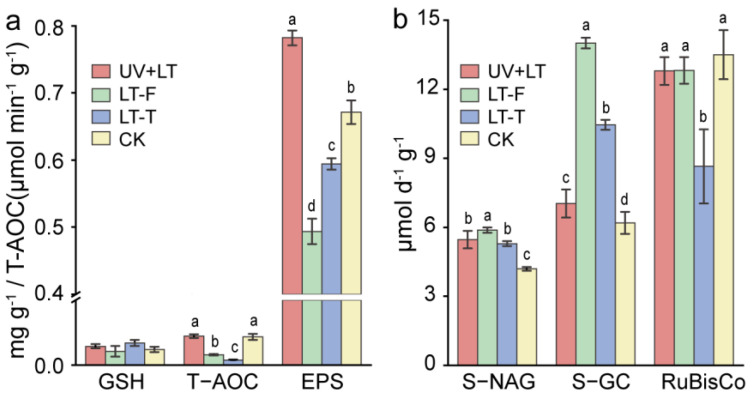
Stress resistance ability (**a**) and carbon metabolism enzymes activities (**b**) on cyanobacterial crusts. GSH, glutathione; T−AOC, total antioxidant capacity; EPS, exopolysaccharide; S−NAG, soil N-cetyl-D-lucosidase; S−GC, soil β-lucosidase; RuBisCo, ribulose-isphosphate carboxylase. UV + LT: stratospheric exposure; LT-F: low-temperature freezing; LT-T: low-temperature thawing; CK: natural control. Error bars show mean ± SD and different lowercase letters indicate significant difference (Tukey HSD; *p* < 0.05) between treatments and control (*n* = 3).

Total antioxidant capacity (T−AOC) was significantly lower in both low-temperature treatment groups than in the control group, whereas T−AOC was not significantly different between the UV + LT treatment group and the control group (F = 143.62, *p* < 0.001). The antioxidant GSH exhibited no significant difference between the three treatment groups and the control group (Figure 3a). EPS content significantly increased by 16% after the UV + LT treatment and reduced in both low-temperature treatment groups, compared with that in the control group (F = 207.841, *p* < 0.001). The activity of RuBisCo was significantly lower in the LT-T treatment group, but no significance was observed in any other treatment groups (F = 13.38, *p* = 0.002). The activities of two important enzymes, S−NAG and S−GC, respectively involved in the degradation of cellulose and chitin, were significantly higher in all three treatment groups (S−NAG: 26~30%; F = 35.59, *p* < 0.001. S−GC: 14~126%; F = 219.80, *p* < 0.001). However, the S−NAG activity under UV + LT and LT-T treatments was elevated less than that under the LT-F treatment, and the elevation of S−GC activity was lowest in the UV + LT treatment (Figure 3b).

### 3.2. Changes in Bacteria Community

A total of 1294 and 1127 OTUs were obtained from DNA and RNA, respectively. DNA and RNA sequencing results showed no significant differences in bacterial Shannon diversity index between the three treatment groups and the control group. However, RNA sequencing indicated a significant elevation in Chao1 index (F = 7.72, *p* = 0.01) under the LT-T treatment (Table 1).

**Figure 4:**
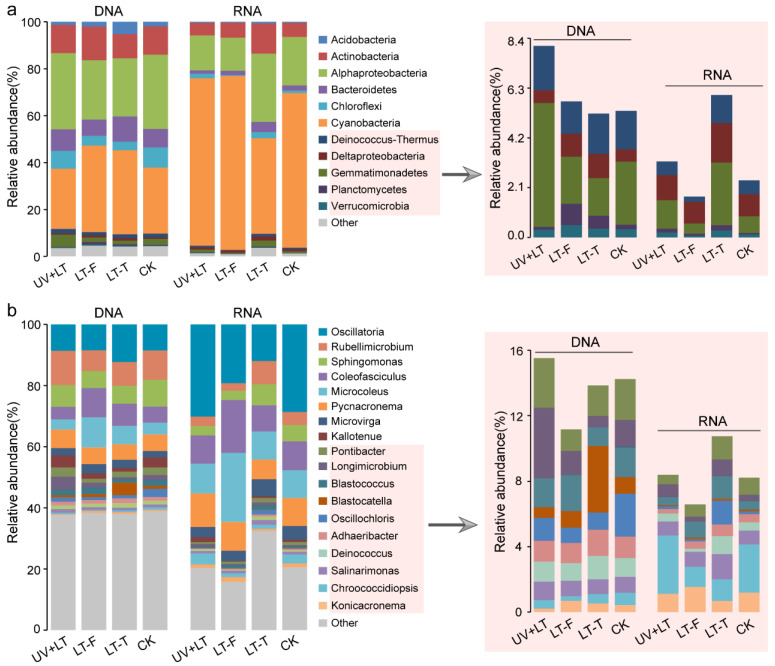
Bacteria community composition on phylum (or class for the Proteobacteria) (**a**) and genus (**b**) level. The right figures show the relative abundance of taxa in the mistyrose box. UV + LT: stratospheric exposure; LT-F: low-temperature freezing; LT-T: low-temperature thawing; CK: natural control.

The DNA and RNA sequences were annotated against the non-redundant nucleotide database and clustered into 18 and 14 phyla, respectively. The relative abundances of 10 phyla were higher than 0.1%. Among them, Cyanobacteria (DNA: 25.69–36.85%, RNA: 40.69–74.14%), Proteobacteria (DNA: 27.06–33.48%, RNA: 15.41–31.84%), and Actinobacteria (DNA: 10.24–14.29%; RNA: 5.40–12.78%) were the three most abundant phyla (Figure 4a). Among the proteobacterial classes, Alphaproteobacteria was the most abundant class (DNA: 24.83–32.41%, RNA: 14.11–29.10%). DNA and RNA sequencing results showed different changing trends in the composition of bacterial communities. At the DNA level, the relative abundance of Gemmatimonadetes in the UV + LT group was about twice as high as that in the control group. The relative abundances of Cyanobacteria, Deltaproteobacteria, and Planctomycetes increased in both low-temperature treatment groups, while those of Alphaproteobacteria and Chloroflexi decreased. At the RNA level, the relative abundances of Chloroflexi and Gemmatimonadetes doubled in the UV + T treatment group, while those of Alphaproteobacteria and Bacteroidetes decreased by one-third. The relative abundances of many taxa decreased in the LT-F treatment group, while a few taxa exhibited a slight increase, such as Cyanobacteria. However, the relative abundances of most taxa substantially increased in the LT-T treatment group, and only that of Cyanobacteria declined in this group. These changes in RNA-based bacterial communities’ structure corresponded to changes in the content of Chl-a and Bchl.

The relative abundances of different genera (average relative abundance > 0.8%) among the three treatments showed obvious differences (Figure 4b). At the DNA level, *Oscillatoria* (8.41–12.28%) and *Rubellimicrobium* (6.82–11.19%) were the two most abundant genera. The relative abundance of *Oscillatoria* was increased under the LT-T treatment, and that of *Rubellimicrobium* was increased under the UV + LT treatment but decreased under the two low-temperature treatments. The relative abundances of *Sphingomonas*, *Oscillochloris*, and *Chroococcidiopsis* were decreased under the three treatments (UV + LT, LT-T, and LT-F), whereas that of *Microvirga* was increased under these three treatments. At the RNA level, *Oscillatoria* (11.95–30.16%), *Coleofasciculus* (8.66–17.34%), *Microcoleus* (9.05–22.56%), and *Pycnacronema* (6.43–10.98%) were the most abundant genera, and their relative abundances changed only slightly in the UV + LT treatment group, compared with that in the control group. The relative abundances of *Kallotenue* and *Longimicrobium* were nearly doubled under the UV + LT treatment, whereas those of *Sphingomonas*, *Pontibacter*, and *Adhaeribacter* were decreased approximately by halved. Under both low-temperature treatments, only the relative abundance of *Blastococcus* was increased, while those of *Oscillatoria*, *Chroococcidiopsis*, and *Kallotenue* were decreased. Eleven genera exhibited the opposite pattern between the two low-temperature treatments, especially *Oscillochloris*. *Oscillochloris* was decreased under the LT-F treatment but increased sharply under the LT-T treatment.

### 3.3. Changes in Relative Abundance of Archaea

**Figure 5:**
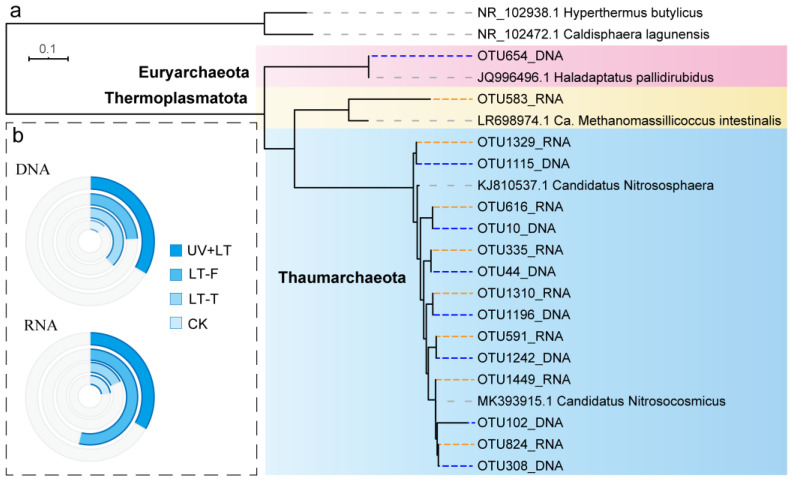
The neighbor-jointing tree (**a**) and relative abundance (**b**) of archaea. The blue and the gray in the circle represent Thaumarchaeota and other taxa, respectively. The larger the radian, the higher the relative abundance. The gray, bule and orange dot lines in the tree represent reference sequences, DNA sequences and RNA sequences, respectively. UV + LT: stratospheric exposure; LT-F: low-temperature freezing; LT-T: low-temperature thawing; CK: natural control.

A total of 1314 and 1491 OTUs were detected from archaea via DNA and RNA amplification sequencing, respectively. In these sequences, only a few archaeal OTUs could be annotated to known taxa. Most of the annotated taxa were Thaumarchaeota (subclassified into Nitrososphaeraceae), and only two OTUs with their relative abundances below 0.01% were annotated as Euryarchaeota and Thermoplasmatota, respectively (Figure 5a). At the DNA level, the relative abundance of Nitrososphaeraceae was increased by 1.9~3.0-fold under the three treatments (Figure 5b). At the RNA level, the relative abundance of Nitrososphaeraceae was increased under UV + LT and LT-F treatments, and it nearly doubled under the LT-F treatment (Figure 5b).

## 4. Discussion

### 4.1. Damage Degree and Tolerance Mechanisms of Cyanobacterial Crusts

In this study, the determination results of total antioxidant capacity, carotenoid, and glutathione content indicated that cyanobacterial crusts microbial communities suffered less UV damage in the stratosphere. UV radiation in the stratosphere (UVB dose of 15.36 kJ m^−2^ or 89.86 kJ m^−2^) was found to be more damaging to microorganisms than reduced atmospheric pressure, high desiccation, and low temperatures (−56.5 °C or −70 °C) [14,38]. After adding 1 mm of Martian mineral analogs to the surface of *Chroococcidiopsis* cells, the damage to photosynthetic pigments in the cells was weak even after 4 h exposure to UVC (53.71 KJ m^−2^) [39]. After 99 h (UV 200~400 nm: 5 × 10^5^ KJ m^−2^) exposure to a Mars-like environment in the form of biofilms, the damage to the lower-layer *Chroococcidiopsis* cells was weak by UV radiation due to the existence of EPS in biofilms [40]. Taken together, both topsoil particles and EPS contributed to attenuating UV radiation damage. EPS was produced when crusts microorganisms were active [41]. Although the real-time temperature in the stratosphere was below 0 °C, cyanobacterial crusts were able to absorb heat from solar radiation, thus increasing the soil temperature. Studies have shown that soil microorganisms in the Qinghai–Tibetan Plateau, Taklimakan Desert, Mu Us Desert, and Tengger Desert still have aerobic respiration activity under −10 °C~0 °C in winter [42,43,44,45]. Although the stratospheric environment was different from that on the ground, our results show that crusts microorganisms exhibited activity at a certain period of time during stratospheric exposure. In addition, the antioxidant enzymes activities of the desert cyanobacterium *Scytonema javanicum* and *Phormidium tenue* were increased after 30 min of UVB radiation [20,21], indicating that crusts microorganisms were able to quickly respond to stressful environments. In our stratospheric exposure experiment (UVB dose of about 56.45 kJ m^−2^, UVC dose of 1.87 kJ m^−2^), the content of EPS was significantly increased (Figure 3a). EPS can not only reduce radiation intensity but also scavenge reactive oxygen species (ROS) [46,47]. ROS are produced in large quantities under the induction of UV radiation, thus resulting in DNA strand breakage, protein conformation change, and biofilm peroxidation [48,49]. Therefore, cyanobacterial crusts microorganisms could resist UV stress in the stratosphere through EPS.

The simulated freeze–thaw experiment showed that the changing low-temperature damaged CCs, especially under slow thawing treatment (Figure 2 and Figure 3). The changes in carbon-degrading enzyme activities indicated that cyanobacterial crusts resisted extreme low-temperature stress most probably by increasing the content of monosaccharides. Low temperature (below 0 °C) will cause intracellular and extracellular damage to cells. In order to increase the concentration of cell fluid to reduce the freezing point, plant cells can regulate their osmotic potential by producing a variety of substances, such as sucrose, fructose, and glucose [50,51]. For example, the soluble monosaccharide content in *Syntrichia caninervis* Mitt. of moss crusts was significantly increased after freeze–thaw cycles [52]. During freeze–thaw cycles, slow freezing contributes to maintaining intracellular and extracellular osmotic equilibrium, thus limiting cell damage. However, slow thawing could cause continuous recrystallization of ice crystals inside cells, leading to cell death and increasing soil respiration rates [53]. We also observed the greater damage caused by simulated low-temperature treatments than by stratospheric light exposure. There were two possible explanations for this difference: (1) In the stratospheric exposure experiment, gradual thawing was accompanied by an increase in light intensity, thus possibly reducing the damage by slow thawing; (2) the environment was drier in the stratosphere than in the laboratory, and the decline in water content in the crusts exposed to stratosphere may limit the damage caused by freeze–thaw cycling.

### 4.2. Regulation of Microbial Communities by Stratospheric Environment

Our data indicated significant differences in the community structures between the DNA and RNA levels, which was consistent with previous studies. Through 16S rRNA gene (DNA) sequencing, the total community composition was obtained, including both active and dormant cells [54]. This sequencing also captured extracellular DNA that was preserved in the soil by either adsorption to soil particles or as components of the extracellular polymeric substances [55,56]. In contrast, 16S rRNA (RNA) sequencing captured indicative of the active members of the microbial communities [57]. RNA is stable only inside the cell, and extracellular RNA is easily and rapidly degraded. Moreover, RNA responds more quickly to changing environmental conditions [58].

At the RNA level, microbial community composition results showed that Chloroflexi and Gemmatimonadetes were more resistant to the extreme stratospheric environment than Cyanobacteria, especially *Kallotenue* and *Longimicrobium* (Figure 4). Chloroflexi is a dominant bacterial phylum in many extreme environments, such as alkaline hot springs, deep lakes, and arctic soils [59,60,61]. Chloroflexi can be photoautotrophic, chemoautotrophic, or mixotrophic, and they can adapt their metabolic strategy depending on the prevailing environmental conditions [62,63]. *Kallotenue* (belonging to Chloroflexi) could hydrolyze cellulose into glucose [64]. Our enzyme activity experiments also indicated that microorganisms could resist the stratospheric extreme conditions by degrading cellulose (Figure 3). Thus, *Kallotenue* was well adapted to these extreme conditions. Gemmatimonadetes can survive in environments with low soil moisture (<15%) [65], and they potentially oxidized hydrogen, carbon monoxide, and methane to produce energy under a lack of organic carbon [66]. These characteristics might be helpful to the improvement of survival rates of Gemmatimonadetes in the stratosphere. *Longimicrobium* (belonging to Gemmatimonadetes) can excrete granular substances, which adsorbs to the cell surface, thereby leading to cellular aggregation [67]. This helps internal cells to resist extreme conditions. Nitrososphaeraceae can oxidize ammonia and generate energy [68], which might make them superior to other archaea in survival under extreme conditions. Microorganisms with diverse metabolic strategies are more likely to tolerate stratospheric extreme environments.

Our simulation experiment results showed that low temperature in the stratosphere could change the microbial community structure. By analyzing the variation in microbial abundance at the RNA level, we found that *Blastococcus* was tolerant to freeze–thaw stress. The predominant cellular fatty acids of various *Blastococcus* strains have been reported to be unsaturated and iso-branched fatty acids [69,70], which is conducive to a reduction in the critical temperature at which cell membranes are transformed into the gel phase [71], thereby conferring the microorganisms with better resistance to low-temperature stress. Besides, two common aerobic photosynthetic bacteria (AAPB) *Oscillochloris* and *Sphingomonas* [72,73], are tolerant to the thawing treatment, especially *Oscillochloris*. This explains the increase in bacterial chlorophyll content under the low-temperature thawing treatment. Previous studies revealed that *Oscillochloris* was mesophilic bacteria [74,75], whereas our data indicated that *Oscillochloris* were able to tolerate low-temperature below zero in cyanobacterial crusts, which mighty be related to the unique property of *Oscillochloris* in crusts. *Sphingomonas* is the main AAPB in Antarctica, and a variety of *Sphingomonas* strains have the ability to adapt to extreme cold [76,77,78].

## 5. Conclusions

This study made the first attempt to expose microbial communities (represented by cyanobacterial crusts microbial communities) to the stratosphere. After 140 min of light exposure, the majority of species showed slight changes in RNA abundance, and *Kallotenue* and *Longimicrobium* were the most tolerant genera. Two genera, *Blastococcus* and *Oscillochloris*, exhibited an extremely high tolerance to low temperature in simulated stratospheric freeze–thaw cycles. The metabolic activity measurements revealed that short-term stratospheric exposure resulted in weak oxidative damage and a slight impact on the photosynthetic activity of cyanobacterial crusts. Microbial degradation of cellulose and chitin was one of the mechanisms by which crusts resist extreme stratospheric conditions. Our results expand the understanding of the tolerance in crusts microbial communities and provide a new insight into the mechanisms of resistance to extreme conditions. Whether microorganisms in cyanobacterial crusts can photosynthesize in the stratosphere remains to be further investigated.

## Figures and Tables

**Table 1:** The alpha diversity indexes of bacteria and archaea based on the OTUs data.

		DNA				RNA			
		UV + LT	LT-F	LT-T	CK	UV + LT	LT-F	LT-T	CK
Bacterial	OTUs	812 ± 16	844 ± 74	859 ± 24	877 ± 8	618 ± 77 b	565 ± 38 b	749 ± 59 a	582 ± 66 b
	Shannon	4.69 ± 0.23	4.67 ± 0.15	4.59 ± 0.27	4.72 ± 0.19	3.75 ± 0.49	3.65 ± 0.35	4.42 ± 0.45	3.82 ± 0.60
	Chao1	947 ± 58	1040 ± 65	975 ± 23	953 ± 59	777 ± 84 b	739 ± 16 b	912 ± 49 a	739 ± 29 b
Archaea	OTUs	499 ± 107	664 ± 178	592 ± 225	559 ± 158	611 ± 309	500 ± 323	457 ± 312	800 ± 88
	Shannon	3.24 ± 0.54	3.89 ± 0.87	3.49 ± 1.04	4.09 ± 0.62	3.63 ± 1.31	3.05 ± 1.30	3.72 ± 0.49	4.52 ± 0.54
	Chao1	670 ± 78	831 ± 200	734 ± 222	721 ± 140	787 ± 281	665 ± 340	681 ± 367	983 ± 67

Values are mean ± standard error. Different lowercase letters represent significant difference (Tukey HSD; *p* < 0.05) between treatments and control (*n* = 3); UV + LT: stratospheric exposure; LT-F: low-temperature freezing; LT-T: low-temperature thawing; CK: natural control.

## Data Availability

The bacterial and archaea raw sequences were submitted to NCBI, and their accession were PRJNA703425 and PRJNA703769, respectively.

## Supplementary Materials

The following supporting information can be downloaded at: https://www.mdpi.com/article/10.3390/microorganisms10061252/s1, Figure S1: The detailed structure of the sample box. Table S1: The real-time temperature and light intensity in the flight experiment.
