# Supplementary figures and images for: Responses of Cyanobacterial Crusts and Microbial Communities to Extreme Environments of the Stratosphere
Source: Microorganisms. 2022 Jun 19;10(6):1252. doi: 10.3390/microorganisms10061252 (PMC9230428; doi:10.3390/microorganisms10061252)

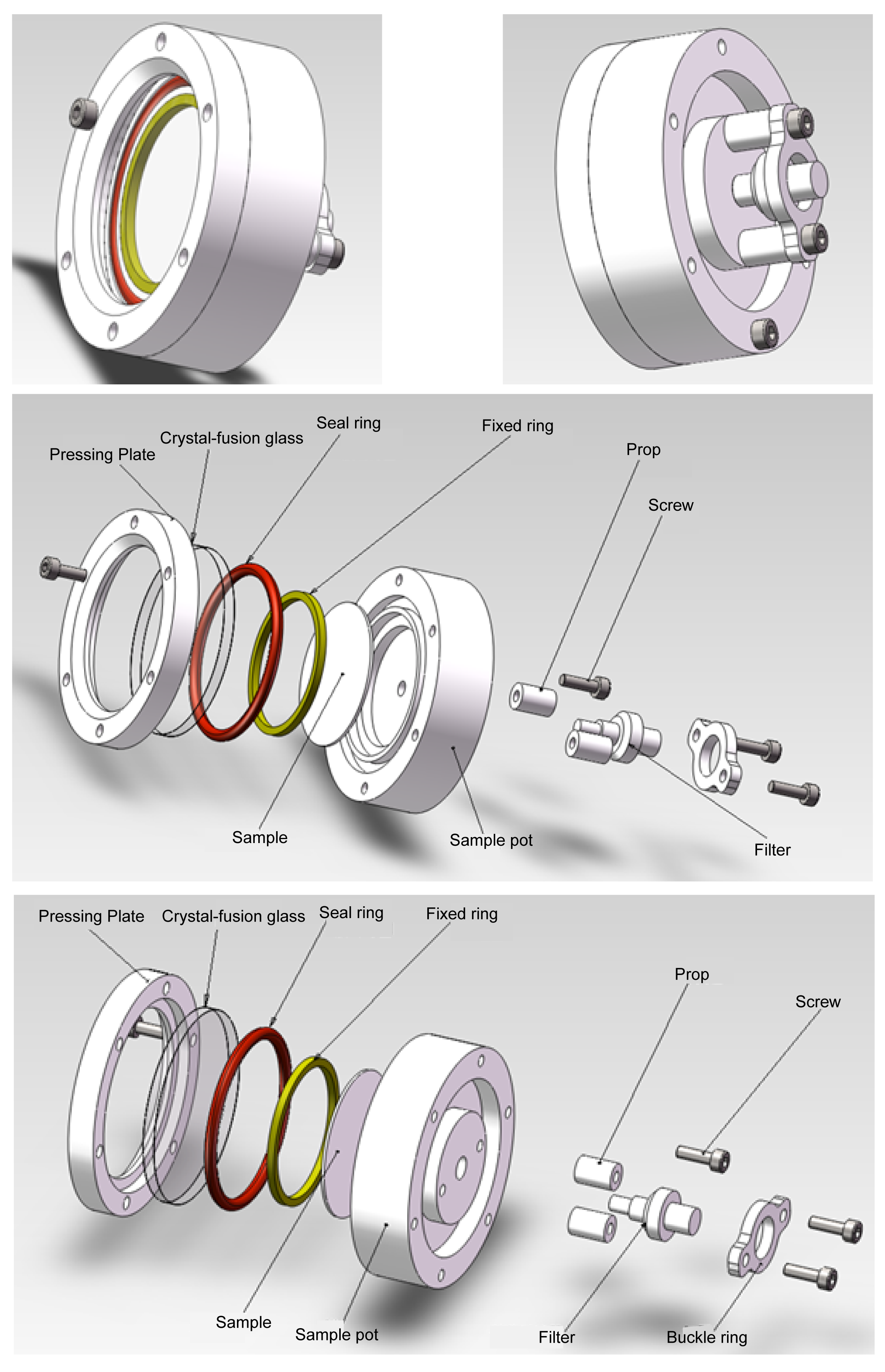

Supplement: Supplementary file 1 [file microorganisms-10-01252-s001.zip › Figure S1.tif]
